# Supplementary material for: Novel Driver Strength Index highlights important cancer genes in TCGA PanCanAtlas patients
Source: PeerJ. 2022 Aug 11;10:e13860. doi: 10.7717/peerj.13860 (PMC9375969; doi:10.7717/peerj.13860)
Supplement: Supplemental Information 2 [file peerj-10-13860-s002.zip › COHORTS/genes plots/detailed/2021_8_16_14_9_distribution_events_detailed_females_42.pdf]

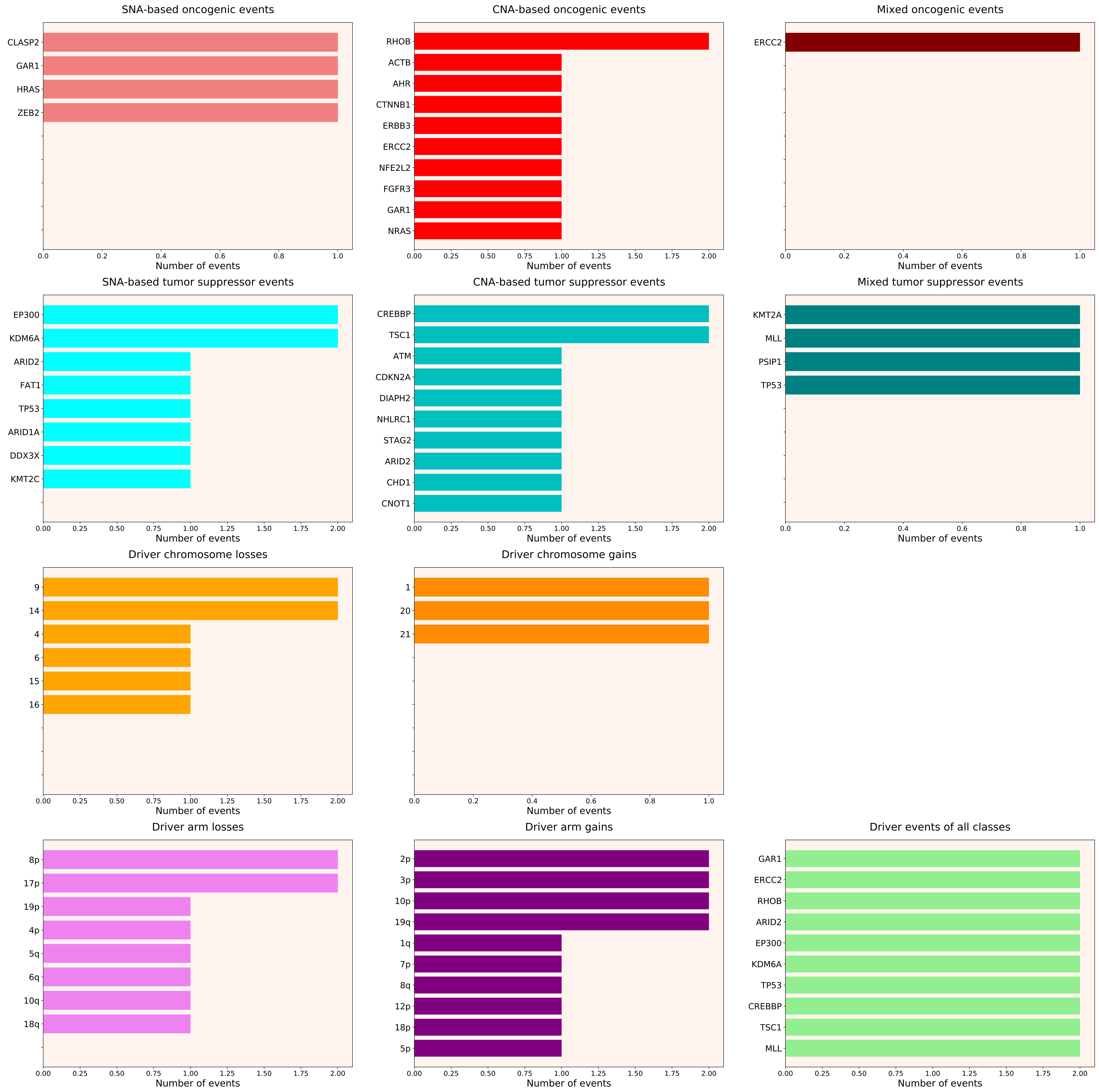
SNA-based oncogenic events
CLASP2
GAR1
HRAS
ZEB2
Number of events
CNA-based oncogenic events
RHOB
ACTB
AHR
CTNNB1
ERBB3
ERCC2
NFE2L2
FGFR3
GAR1
NRAS
Number of events
Mixed oncogenic events
ERCC2
Number of events
SNA-based tumor suppressor events
EP300
KDM6A
ARID2
FAT1
TP53
ARID1A
DDX3X
KMT2C
Number of events
CNA-based tumor suppressor events
CREBBP
TSC1
ATM
CDKN2A
DIAPH2
NHLRC1
STAG2
ARID2
CHD1
CNOT1
Number of events
Mixed tumor suppressor events
KMT2A
MLL
PSIP1
TP53
Number of events
Driver chromosome losses
9
14
4
6
15
16
Number of events
Driver chromosome gains
1
20
21
Number of events
Driver arm losses
8p
17p
19p
4p
5q
6q
10q
18q
Number of events
Driver arm gains
2p
3p
10p
19q
1q
7p
8q
12p
18p
5p
Number of events
Driver events of all classes
GAR1
ERCC2
RHOB
ARID2
EP300
KDM6A
TP53
CREBBP
TSC1
MLL
Number of events
